# Evolutionary and Biogeographic Insights on the Macaronesian *Beta*-*Patellifolia* Species (Amaranthaceae) from a Time-Scaled Molecular Phylogeny

**DOI:** 10.1371/journal.pone.0152456

**Published:** 2016-03-31

**Authors:** Maria M. Romeiras, Ana Vieira, Diogo N. Silva, Monica Moura, Arnoldo Santos-Guerra, Dora Batista, Maria Cristina Duarte, Octávio S. Paulo

**Affiliations:** 1 Biosystems and Integrative Sciences Institute (BioISI), Faculdade de Ciências, Universidade de Lisboa, Lisbon, Portugal; 2 Centre for Ecology, Evolution and Environmental Changes (CE3C), Faculdade de Ciências, Universidade de Lisboa, Lisbon, Portugal; 3 Centro de Investigação das Ferrugens do Cafeeiro, Instituto Superior de Agronomia, Universidade de Lisboa, Oeiras, Portugal; 4 Research Network in Biodiversity and Evolutionary Biology, Associate Laboratory (CIBIO/InBIO), Universidade do Porto, Vairão, Portugal; 5 Universidade dos Açores, Dep. Biologia, Ponta Delgada, Portugal; 6 Unidad de Botánica-Jardín de Aclimatación de La Orotava (ICIA), Tenerife, Spain; Muséum national d'Histoire naturelle, FRANCE

## Abstract

The Western Mediterranean Region and Macaronesian Islands are one of the top biodiversity hotspots of Europe, containing a significant native genetic diversity of global value among the Crop Wild Relatives (CWR). Sugar beet is the primary crop of the genus *Beta* (subfamily Betoideae, Amaranthaceae) and despite the great economic importance of this genus, and of the close relative *Patellifolia* species, a reconstruction of their evolutionary history is still lacking. We analyzed nrDNA (ITS) and cpDNA gene (*matK*, *trnH-psbA*, *trnL intron*, *rbcL*) sequences to: (i) investigate the phylogenetic relationships within the Betoideae subfamily, and (ii) elucidate the historical biogeography of wild beet species in the Western Mediterranean Region, including the Macaronesian Islands. The results support the Betoideae as a monophyletic group (excluding the *Acroglochin* genus) and provide a detailed inference of relationships within this subfamily, revealing: (i) a deep genetic differentiation between *Beta* and *Patellifolia* species, which may have occurred in Late Oligocene; and (ii) the occurrence of a West-East genetic divergence within *Beta*, indicating that the Mediterranean species probably differentiated by the end of the Miocene. This was interpreted as a signature of species radiation induced by dramatic habitat changes during the Messinian Salinity Crisis (MSC, 5.96–5.33 Mya). Moreover, colonization events during the Pleistocene also played a role in shaping the current diversity patterns among and within the Macaronesian Islands. The origin and number of these events could not be revealed due to insufficient phylogenetic resolution, suggesting that the diversification was quite recent in these archipelagos, and unravelling potential complex biogeographic patterns with hybridization and gene flow playing an important role. Finally, three evolutionary lineages were identified corresponding to major gene pools of sugar beet wild relatives, which provide useful information for establishing *in situ* and *ex situ* conservation priorities in the hotspot area of the Macaronesian Islands.

## Introduction

The potentially devastating impact of climate change on biodiversity and food security, together with the growing world population, means that taking action to conserve Crop Wild Relative (CWR) diversity is an urgent priority [1]. CWR tend to contain greater genetic variation than their respectively derived crops because they have not endured the genetic bottleneck of domestication [2]. The contribution of CWR to improve crop performance is growing and has largely been achieved through the donation of useful genes for pest and disease resistance, and abiotic stress tolerance [3,4]. Although, in the light of contemporary biotechnological advances, most crop wild relative species are potential gene donors to a crop, it is essential to understand how closely a taxon is related to a crop for it to be considered a CWR [2]. The gene pool concept [5] proposes that members of crop primary gene pool (GP1) and of secondary gene pool (GP2) are most likely to be crossable with the crop; but CWR in the tertiary gene pool (GP3) require biotechnological approaches to facilitate gene transfer. The application of the gene pool concept provides a pragmatic way of establishing the degree of CWR relatedness and thus assists in establishing conservation priorities [6].

The Euro-Mediterranean Region has a significant genetic diversity of global value both in crops of major socio-economic importance and their wild relatives, such as oats (*Avena sativa* L.), carrots (*Daucus carota* L.), apples (*Malus domestica* Borkh.) and sugar beet (*Beta vulgaris* L. subsp. *vulgaris*) (e.g. [7]). Sugar beet is the primary crop of the genus *Beta* L. and is the most economically valuable crop species in the Order Caryophyllales [8]. Sugar beet provides around one third of the sugar consumed worldwide and serves as a significant source of bioenergy in the form of ethanol [9]. *Beta vulgaris* subsp. *vulgaris* also includes crop types used as root and leafy vegetables (e.g. beetroot, Swiss chard) to provide both food and fodder since ancient times, and all are ultimately derived from the wild sea beet (*B*. *vulgaris* subsp. *maritima* (L.) Arcang.), which is predominantly found in coastal areas around and adjacent to the Mediterranean Sea [10].

Beet (*Beta vulgaris s*.*l*.) is included in the small subfamily Betoideae Ulbr. (Amaranthaceae family), which comprises six genera: *Beta* L., *Patellifolia* A.J.Scott, Ford-Lloyd & J.T.Williams, and the monotypic genera *Acroglochin* Schrad., *Aphanisma* Nutt. ex Moq., *Hablitzia* M.Bieb. and *Oreobliton* Durieu & Moq. Among the total number of fourteen taxa considered in the taxonomy of *Beta s*.*l*. [10,11], seven are commonly found in coastal areas of the Western Mediterranean Region and the Macaronesian Islands: *B*. *macrocarpa* Guss., *B*. *patula* Aiton, and *B*. *vulgaris* subsp. *maritima* and subsp. *vulgaris* (from section *Beta*); and *Patellifolia patellaris* (Moq.) A.J.Scott, Ford-Lloyd & J.T.Williams, *P*. *procumbens* (C.Sm.) A.J.Scott, Ford-Lloyd & J.T.Williams, and *P*. *webbiana* (Moq.) A.J.Scott, Ford-Lloyd & J.T.Williams (formerly included in section *Procumbentes* of the genus *Beta*). The remaining beet species occur in the Eastern Mediterranean Region and Southwestern Asia (i.e. *B*. *corolliflora* Zosimovic ex Buttler, *B*. *intermedia* Bunge ex Boiss., *B*. *lomatogona* Fisch. & C.A.Mey., *B*. *macrorhiza* Steven, and *B*. *trigyna* Waldst. & Kit. from section Corollinae; and *B*. *nana* Boiss. & Heldr. from section Nanae) (see Table 1, showing species distribution, ecology, and IUCN conservation status).

**Table 1:** Native geographical distribution, ecology, and IUCN conservation status of taxa from subfamily Betoideae; taxonomy according to Kadereit et al. [16].

Taxon	Geographical distribution ^a^					Ecology ^a^	Conservation status
	Macaronesia				Worldwide		
	Azores	Madeira	Canaries	Cape Verde			
***Acroglochin* Schrad.**^b^							
*Acroglochin persicarioides (Poir.) Moq. (inc. *A*. *obtusifolia* C.H.Blom)							
					Bhutan, China (Yunnan), Nepal, N Pakistan, India (Himalayas)	Forest margins, riversides, open hillsides, fields, roadsides, wastelands	Not Evaluated
***Aphanisma* Nutt. ex Moq.**							
*Aphanisma blitoides Nutt. ex Moq.							
					N America (California, Mexico)	Coastal shrublands, coastal bluffs, saline sands, sand dunes	Vulnerable^c^
***Beta* L.**							
**section *Beta***							
**Beta macrocarpa Guss.							
			La Palma; Tenerife; Gran Canaria; Fuerteventura; Lanzarote		SW (Portugal, Spain) and SE (Greece, Italy) Europe, N Africa, W Asia, Macaronesia	Dry coastal sites, salt marshes, salt pans, field margins, along roadsides	Endangered^c^
**Beta patula Aiton							
		Madeira (Ilhéu do Desembarcadouro; Ilhéu de Fora); Desertas (Ilhéu Chão)			Macaronesia	Dry coastal sites	Critically Endangered^d^
*Beta vulgaris* subsp. *adanensis* (Pamukç.) Ford-Lloyd & J.T.Williams							
					SE Europe (Greece), W Asia (Cyprus, Turkey, Syria)	Disturbed habitats, steppes	Not Evaluated
**Beta vulgaris subsp. *maritima* (L.) Arcang.							
	Faial; Pico; Graciosa; S. Jorge; Terceira; S. Miguel; S. Maria	Madeira; Porto Santo; Desertas	El Hierro; La Palma; La Gomera; Tenerife; Gran Canaria; Fuerteventura; Lanzarote		N, SW and SE Europe (Atlantic coasts), Mediterranean coasts, N Africa, Macaronesia, W Asia	Coastal cliffs, stony and sand beaches, salt marshes, saline explorations, coastal grasslands, ruderal places	Least Concern^c^
**Beta vulgaris subsp. *vulgaris*							
					Widely cultivated		
**section *Corollinae* Ulbr.**							
*Beta corolliflora Zosimovic ex Buttler							
					SW Asia, Caucasus	Over 1300 m altitude in ruderal places, meadows, pastures, stream banks	Not Evaluated
*Beta lomatogona* Fisch. & C.A.Mey.							
					Turkey, Iran, Azerbaijan, Armenia	Mountain areas (850–1500 m altitude), saline areas, steppes, semi-deserts, dry wastelands, roadsides, agricultural land	Not Evaluated (EN—in Armenia)
*Beta macrorhiza* Steven							
					Armenia, Azerbaijan, Dagestan, Turkey	Steppes, agricultural land, dry river beds, wastelands in mountainous regions	Not Evaluated
*Beta nana Boiss. & Heldr.							
					SE Europe (Greece)	Ruderal places, grassland soil depressions over 1800 m altitude	Vulnerable^d^
*Beta trigyna Waldst. & Kit.							
					W Asia, Caucasus, E and SE Europe	Ruderal places, grasslands	Data Deficient^c^
*Beta intermedia* Bunge ex Boiss. (probably a hybrid of *B*. *lomatogona × B*. *trigyna*) ^e^							
					Armenia, Turkey		Not Evaluated
***Hablitzia* M.Bieb.**							
*Hablitzia tamnoides M.Bieb.							
					Caucasus (Armenia, Azerbaijan, Georgia, Russia—Ciscaucasia, Dagestan)	Forests areas	Not Evaluated
***Oreobliton* Durieu & Moq.**							
*Oreobliton thesioides Durieu & Moq.							
					N Africa (Algeria, Tunisia)	Chalk rocks in the Atlas mountains	Not Evaluated
***Patellifolia* A.J.Scott, Ford-Lloyd & J.T.Williams**							
**Patellifolia patellaris (Moq.) A.J.Scott, Ford-Lloyd & J.T.Williams							
		Madeira; Porto Santo; Salvages	El Hierro; La Palma; La Gomera; Tenerife; Gran Canaria; Fuerteventura; Lanzarote	Santo Antão; São Vicente	Iberian Peninsula, SE Europe (Italy), N Africa, Macaronesia	Dry coastal sites, low-lying dry rock areas	Least Concern^c^
**Patellifolia procumbens (C.Sm.) A.J.Scott, Ford-Lloyd & J.T.Williams							
		Madeira (Ilhéu do Desembarcadouro); Desertas (Ilhéu Chão); Porto Santo (Ilhéu de Fora); Salvages	El Hierro; La Palma; La Gomera; Tenerife; Gran Canaria; Fuerteventura; Lanzarote	Santo Antão; São Vicente; São Nicolau; Boavista; Maio; Santiago	Macaronesia	Dry coastal sites	Least Concern^c^
**Patellifolia webbiana (Moq.) A.J.Scott, Ford-Lloyd & J.T.Williams							
			Gran Canaria^f^		Macaronesia	Ruderal nitrophilous sites	Critically Endangered^d^

Taxa marked with an * were included in the phylogenetic analyses; those marked with ** were collected for this study.

^a^ Data from fieldwork and bibliography [23–30]. Permissions to collect protected species from protected areas were issued by Portuguese authorities (Instituto da Conservação da Natureza e das Florestas, ICNF), for Portugal mainland, and Secretaria Regional do Ambiente, for Madeira and Azores), and Cape Verdean authorities (Direcção Geral Ambiente/MAHOT). Material from the Canary Islands was provided by the Orotava Botanical Garden and some of the samples from Madeira were provided by ISOPlexis (Banco de Germoplasma da Universidade da Madeira). For non-protected species specific permissions are not required.

^b^ Classification uncertain within the subfamily Betoideae [16].

^c^ Regional assessment [17, 30].

^d^ Global assessment (http://www.iucnredlist.org/, accessed on 26 June 2015).

^e^ Unresolved name (The Plant List (2015). Version 1.1. Published on the Internet; http://www.theplantlist.org/ (accessed 1st January).

^f^ According to one of the authors (A. Santos-Guerra) this species occurs only in Gran Canaria Island (La Isleta).

The phylogenetic relationships between and within the tribes and sections of the Betoideae subfamily are still far from resolved especially with regard to the acceptance of the genus *Patellifolia*. The latter differs from *Beta* by having short tepals that do not overtop the fruit vs. long tepals that overtop the fruit [12]. Previous studies based on morphological features (e.g. [10,13–14]) failed to recognize *Patellifolia* as a separate genus but rather as part of the *Beta* section *Procumbentes*. Recent molecular phylogenetic studies (e.g. [15,16]) modified the subfamily classification previously proposed. It was also suggested that *Acroglochin* should be excluded from this subfamily and that the other five genera (i.e. *Beta*, *Aphanisma*, *Hablitzia*, *Oreobliton*, and *Patellifolia*) should fall into two clades, i.e. *Beteae* comprising *Beta* only, and *Hablitzieae* with the remaining four genera. These studies have been hampered by the undersampling of species from the Western Mediterranean Region, including the hotspot area of the Macaronesian Islands, where some endemic species are found (i.e. *B*. *patula* in the Madeira archipelago, *P*. *webbiana* in the Canary Islands, and *P*. *procumbens* in all the Macaronesian archipelagos except the Azores). Two of these Macaronesian endemics (i.e. *B*. *patula* and *P*. *webbiana*) were recently classified as Critically Endangered in the European Red List of Vascular Plants [17].

Though the importance of conservation of these wild taxa has been widely recognized [18], it is also important to understand the relationships within the *Beta s*.*l*. gene pools, which will offer an effective approach for utilization of the wild-beet germplasm. For instance it is pointed out that *Patellifolia* species can transmit traits providing resistance to the most serious diseases of sugar beets worldwide, such as sugar beet cyst nematode (*Heterodera schachtii* Schmidt), leaf spot disease caused by *Cercospora beticola* Sacc., curly top virus, rhizomania, and powdery mildew (*Erysiphe polygoni* DC.) [19–22].

Despite the great economic importance of the *Beta* and *Patellifolia* species [18], a reconstruction of the evolutionary history with a dated molecular phylogeny for the subfamily Betoideae is still lacking. The aims of this study are to: (1) present a hypothesis of the phylogenetic relationships within the subfamily Betoideae, and (2) gain a better understanding of the spatio-temporal history of the wild beet species which occur in the hotspot area of Western Mediterranean Region, including for the first time the endemic species from the Macaronesian Islands and samples from all the five archipelagos (i.e. the Azores, Canaries, Cape Verde, Madeira including the Desertas, and Savage Islands).

## Materials and Methods

### Sampling

Among the total number of fourteen taxa included in the subfamily Betoideae, seven are found in coastal areas of the Western Mediterranean Region and the Macaronesian Islands. These taxa belong to the gene pool (GP) of cultivated beets and were purposely sampled for this study [i.e. 43 samples of *Beta*: *B*. *macrocarpa* (7), *B*. *patula* (13), *B*. *vulgaris* subsp. *maritima* (20), and subsp. *vulgaris* (3), all belonging to GP1; and 25 samples of *Patellifolia*: *P*. *patellaris* (11), *P*. *procumbens* (13), *P*. *webbiana* (1), which belong to GP3] (S1 Fig). To include a broad range of species from the Betoideae subfamily, other sequences were obtained from the GenBank. Three *Beta* samples from the Eastern Mediterranean Region and Southwestern Asia were included (i.e. *B*. *corolliflora*, *B*. *nana*, and *B*. *trigyna*, which belong to GP2), and also samples from the 4 monotypic genera: *Aphanisma blitoides* Nutt. ex Moq., from California, *Hablitzia tamnoides* M.Bieb. native to the Caucasus Region, *Oreobliton thesioides* Durieu & Moq., from North-Africa, and *Acroglochin persicarioides* (Poir.) Moq. from remote areas of the Himalayas (Table 1: data from fieldwork and bibliography [23–30]).

Furthermore, nine outgroup species were selected from seven other subfamilies of the Amaranthaceae: (i) Amaranthoideae Burnett (*Amaranthus retroflexus* L.; *Charpentiera obovata* Gaudich.); (ii) Chenopodioideae Burnett (*Atriplex prostrata* Boucher ex DC.); (iii) Corispermoideae Raf. (*Corispermum chinganicum* Iljin); (iv) Polycnemoideae Ulbrich (*Polycnemum majus* A.Braun; *Nitrophila occidentalis* (Moq.) S.Watson); (v) Salicornioideae Luersson (*Arthrocnemum macrostachyum* (Moric.) K.Koch); (vi) Salsoloideae Raf. (*Salsola kali* L.); and (vii) Suaedoideae Ulbr. (*Suaeda maritima* (L.) Dumort.).

GenBank accession numbers are provided in S1 Table for all the studied samples. Additionally, data on sampling sites of the samples collected in this study, including their geographical coordinates, details about vouchers and their respective herbaria, are also included in S1 Table.

### Molecular data

DNA was extracted using DNeasy Plant Mini Kit (QIAGEN, Valencia, California, USA) and purified, using QIAquick columns (QIAGEN, Valencia, California, USA) or Silica Bead DNA Gel Extraction kit (Fermentas), according to the manufacturer’s protocols. Polymerase chain reaction (PCR) amplifications using 20–30ηg of genomic DNA were performed to amplify the complete internal transcribed spacer (ITS) region, using the primers ITS4 and ITS5 [31]. Two coding regions of the chloroplast genome were amplified using primer pairs, *matK* [32] and *rbcL* [33], plus two non-coding regions using *trnL intron* [34] and *trnH—psbA* [35].

PCR amplifications were carried out using a 2720 Thermal Cycler (Perkin-Elmer, Applied Biosystems, Foster City, California, USA) and performed in a final volume of 25μl (1μl of DNA, PCR buffer (20mM Tris-HCl pH 8.4, 50mM KCl), 0.1mg/ml BSA (Ambion), 2mM MgCl2, 0.2mM of each dNTPs, 0.4mM of each primer, and 1 unit Taq DNA polymerase (GibcoBRL)). The PCR cycling scheme was an initial denaturation step at 95°C for 4 min, followed by 40 cycles of denaturation at 94°C for 1 min, annealing depending on the primer pair used at 50°C (*matK*), 55°C (ITS and *rbcL*), 58°C (*trnL intron*) or 65°C (*trnH—psbA*) for 1 min, extension at 72°C for 2 min, followed by a final extension at 72°C for 7 min.

Sixty-eight samples were sequenced for the ITS region (S1 Table). From a preliminary study at population level of the four chloroplast regions, all individuals of each species, of *Beta* and *Patellifolia*, and those from a given sampling location exhibited the same cpDNA sequences. Therefore, cpDNA sequences were generated by selecting a subset of 1–5 individuals/island, resulting in 27 representative accessions sequenced. This sub-sample was generated for *matK*, *trnH-psbA*, *trnL intron* and *rbcL* (S2 Table), but three specimens for the *rbcL* gene and two specimens for the *trnL intron* and *trnH-psbA* spacer could not be sequenced due to PCR amplification problems. Amplified products were purified with Sureclean Plus (Bioline, London, UK) and sent to STAB Vida, Lda (Monte da Caparica, Portugal) for Sanger sequencing. For all the markers, amplicons were sequenced using both directions in ABI 3730 XL DNA Analyzer (Applied Biosystems). Raw sequences were edited and cleaned by hand in SEQUENCHER v4.0.5 (Gene Codes Corporation).

### Phylogenetic analyses

Multiple sequence alignments were built for each locus dataset in MAFFT v6.717b [36], using the L-INS-i method as recommended in the manual for difficult alignments. Each dataset was concatenated into a combined matrix using ElConcatenero [37]. Maximum Likelihood (ML) and Bayesian Inference (BI) methods were used to reconstruct phylogenies from the separate (i.e. ITS, *matK*, *trnH-psbA*, *trnL intron*, and *rbcL*) and combined datasets. ML searches were performed in RAxML v8.0.9 under the GTRGAMMA model with 1000 bootstrap replicates. The best fit model for each locus data set was selected under the AIC, as implemented in MRMODELTEST v2.3 [38] and used in the Bayesian analysis performed in MRBAYES v3.1.2 [39]. Each locus was allowed to have partition-specific substitution parameters. Analyses were generated for 3x10^7^ generations, sampled every 3000^th^ generation and using the default chain heating temperature. The analysis was run three times with one cold and three incrementally heated Metropolis-coupled Monte Carlo Markov chains, starting from random trees. Output files were analyzed and the convergence and mixing of the independent runs were assessed for all parameters using TRACER v1.4 [40]. Trees from the different runs and their associated posterior probabilities (PP) were then combined and summarized in a 50% consensus tree. All computational analyses were performed using the CIPRES Gateway cloud servers [41]. A clade with a PP value > 0.95 or a BS value > 85% was considered well supported. Additionally, for the subfamily Betoideae and using the concatenation of all loci, the NeighborNet algorithm [42] as implemented in SplitsTree v4.0 [43], was used with the default settings to visualize possible incongruences in the dataset. This method relaxes the assumption that evolution follows a strictly bifurcating path and allows for the identification of reticulated evolution or incomplete lineage sorting among the dataset.

Statistics for the alignments and phylogenetic analyses, as well as the model of evolution for the datasets, are presented in S2 Table.

### Divergence time analyses

Divergence times within the subfamily Betoideae were estimated using the Bayesian MCMC algorithm implemented in BEAST v1.7.2 [44]. For this analysis, we used the combination of the ITS and two cpDNA markers (*matK* and *rbcL*), for which outgroup sequences could be obtained. The GTR model of sequence substitution was used for all partitions, except for *rbcL*, which used the GTR+G model. The fossil of *Chenopodipollis multiplex*, from a pollen record found in the United States and dated to the early Paleocene (65–56 Mya), was used to calibrate the root of our phylogenetic tree, which was previously suggested as the best constraint location for the fossil [15]. Therefore, a normal prior was applied to the root of the phylogenetic tree of this study with a mean of 60.5 and a standard deviation of 2, in order to accommodate the fossil age uncertainty (65–56 Mya). A relaxed lognormal molecular clock was used for all partitions, the Yule process was implemented for the tree prior with a constant speciation rate per lineage, and a random tree was used as the starting tree. The Bayesian MCMC was run for 5x10^7^ generations, sampling parameters at every 5000 generations. This analysis was conducted three independent times. Tracer v1.4 [40] was used to assess convergence and correct mixing of all parameters by visually inspecting the log traces and estimating the Effective Sample Size (ESS) of each parameter. Results from the three runs were combined with LogCombiner v1.7.2 [44], after discarding the 10 first % of each analysis as burn-in. The remaining trees were summarized using a Maximum Clade Credibility target tree in TreeAnnotator v1.7.2 [44], as well as Bayesian posterior probability (PP), MEDIAN/ MEAN height and the 95% highest posterior density heights interval (95% HPD) of each node. All computational analyses were performed in the CIPRES Gateway cloud servers [41].

## Results

### Phylogenetic analyses of Betoideae

Here a new phylogeny of the Betoideae is presented based on nuclear (ITS) and cpDNA markers (*matK*, *trnH-psbA*, *trnL intron*, *rbcL*), covering a widespread sampling within this subfamily, and an outgroup information from other plants from Amaranthaceae family (S2 and S3 Figs). The results provide support for the (i) monophyly of this subfamily, with the exclusion of the genus *Acroglochin*; (ii) a deep genetic differentiation between *Beta* and *Patellifolia*, which are monophyletic groups; and (iii) the identification of three monophyletic lineages, corresponding to major gene pools of sugar beet CWR (i.e. GP1, GP2 and GP3).

Maximum Likelihood (ML) and Bayesian Inference (BI) were used to test phylogenetic hypotheses within the Betoideae subfamily. Topology of the ML tree (S2 Fig) obtained using the concatenated ITS and cpDNA markers was similar to that obtained from Bayesian analysis (tree not shown). Both ML and BI found essentially identical tree topologies, revealing the same major clades. The monophyly of the Betoideae is well-supported by the data (BS = 100%; PP = 1), but the monotypic genus *Acroglochin* is excluded from this subfamily. Instead, *Acroglochin persicarioides* constitutes a robust clade (BS = 90%; PP = 1) with *Corispermum chinganicum* (Corispermoideae subfamily), and both are closely related to *Atriplex prostrata* (Chenopodioideae subfamily).

Relationships within the Betoideae subfamily remain somewhat uncertain, since the most basal branches are poorly supported. Therefore, the basal relationships shown among the five genera (i.e. *Aphanisma*, *Beta*, *Hablitzia*, *Oreobliton*, and *Patellifolia*) could be interpreted as polytomic, according to our results. Nevertheless, the close relationship between *Oreobliton* and *Aphanisma* is well-supported (BS = 100%; PP = 1).

Both *Beta* and *Patellifolia* appear to be well-supported monophyletic groups: clade I that includes all samples of the genus *Beta* (BS = 100%; PP = 1), and clade II (BS = 100%; PP = 1), gathering all the *Patellifolia* representatives. Within clade I the *Beta* species found in coastal areas of the Western Mediterranean Region and in the Macaronesian Islands (i.e. *B*. *vulgaris* subsp. *maritima* and subsp. *vulgaris*, *B*. *macrocarpa*, and *B*. *patula*) form a well-supported monophyletic group (BS = 99%; PP = 1), which is sister to the remaining members of *Beta* (i.e. *B*. *corolliflora*, *B*. *nana*, and *B*. *trigyna*) from the Eastern Mediterranean Region (BS = 80%; PP = 1). Moreover, the Macaronesian endemic species, *B*. *patula* (from Madeira) and *P*. *webbiana* (from the Canary Islands) were placed within *Beta* (clade I—together with the other species from the GP1) or *Patellifolia* (clade II—together with the other species from the GP3) clades, respectively. However, our analyses failed to resolve with confidence the relationships among these endemics and the rest of the species resulting in a polytomy (S1 and S2 Figs).

Applying the NeighborNet algorithm to the concatenated dataset reveals a substantial degree of conflicting phylogenetic signal at the divergences of *Beta*, *Patellifolia*, *Hablitzia*, *Aphanisma* and *Oreobliton*. This is evidenced by the substantial number of loops found in these points of the phylogenetic network (S3 Fig). Further loops are found within *Beta* and *Patellifolia* genera, albeit in a smaller number.

### Divergence time analyses

**Fig 1:**
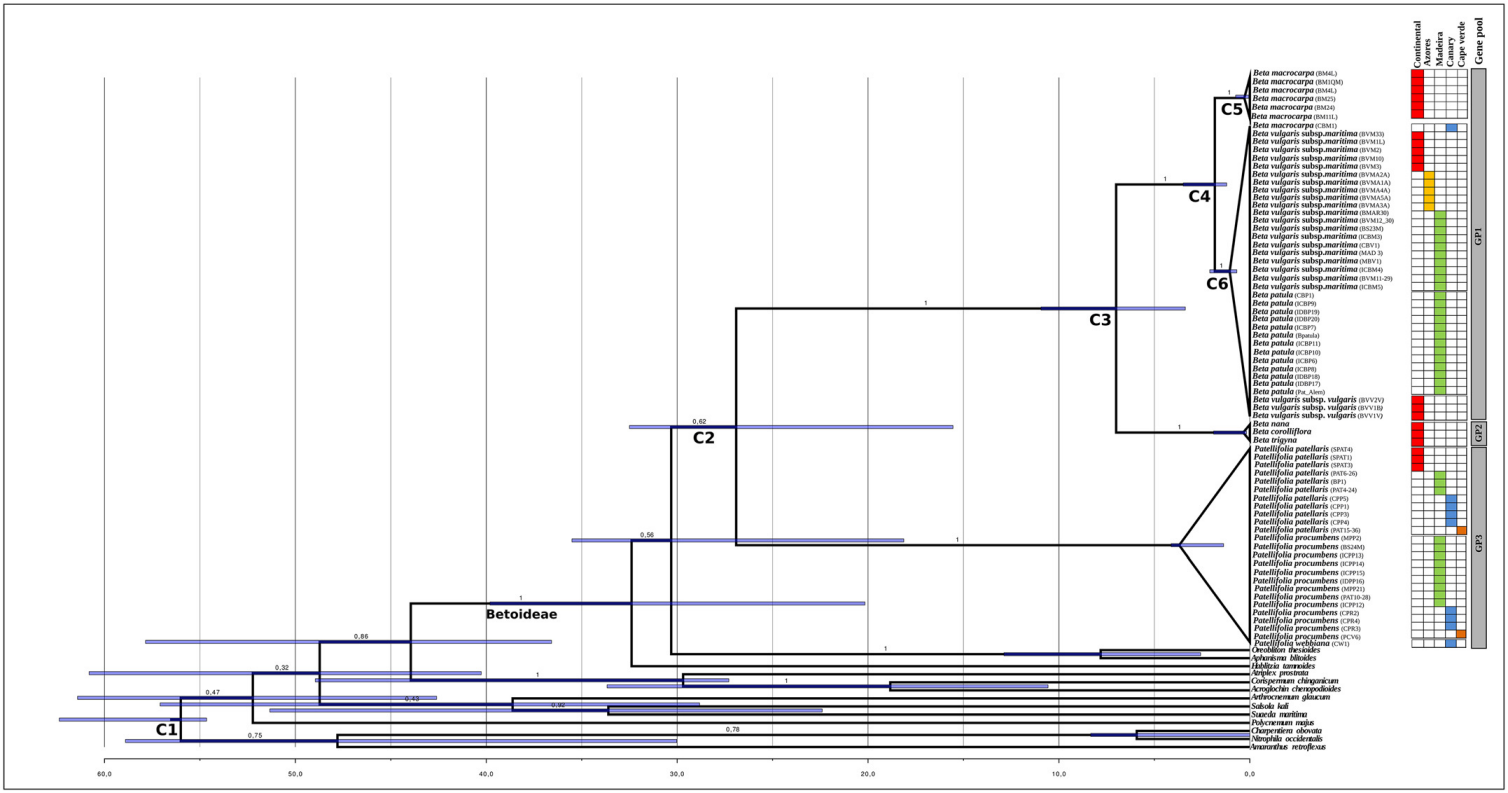
Molecular clock dated phylogenetic tree of Betoideae subfamily (Amaranthaceae). Bayesian tree obtained from the BEAST analysis based on the concatenated dataset of ITS and cpDNA markers (*matK* and *rbcL*), illustrating the estimated divergence ages at selected calibrated nodes. Posterior probabilities (PP) are given above each branch. The geographic origin of each specimen is provided (right side) with a color code for continental areas and several Macaronesian archipelagos and grey bars differentiate the three gene pools previously described (for further details see Frese [10]) and concordant with the present phylogenetic analysis. C1 to C6 as described in Table 2. Mya, million years ago.

Date estimates for nodes within the subfamily Betoideae are presented in Fig 1 (see C1 to C6). Our analysis indicates that the Betoideae must have diverged around 32.5 million years ago (Mya), representing the split between *Hablitzia*, and the other four Betoideae genera. Within this group, the split between *Beta* and *Patellifolia* (C2) was estimated to have occurred at 25.3 Mya (95%HPD: 16.1–34.8 Mya), while *Aphanisma* and *Oreobliton* diverged later at about 8 Mya. In *Beta* group (C3) the Eastern Mediterranean species (i.e. *B*. *corolliflora*, *B*. *nana*, and *B*. *trigyna*) have diverged from the remaining *Beta* species, from the Western Mediterranean and Macaronesian regions, roughly at 7.2 Mya (95%HPD: 3.5–11.5 Mya). Within the Western Mediterranean and Macaronesian species (C4) it was possible to further differentiate a group of *B*. *macrocarpa* sampled from continental regions, that diverged from the remaining *Beta* species at roughly 1.4 Mya (95%HPD: 0.7–2.1 Mya). The latter groups (C5 and C6) have begun their diversification quite recently, probably less than one million years ago (Fig 1 and Table 2)

**Table 2:** Estimation of divergence dates within Betoideae taxa using BEAST as means and 95% highest posterior densities (HPD), in millions of years (Mya).

	Node in Fig 1	Mean age (Mya)	Upper 95% HPD value	Lower 95% HPD value
**C1**	Tree root	60,163	64,191	56,243
**C2**	*Beta* and *Patellifolia* divergence	25,268	34,863	16,066
**C3**	Crown of West/East *Beta* group	7,212	11,532	3,451
**C4**	Crown of West *Beta* group divergence (including *B*. *macrocarpa / B*. *vulgaris* subsp. *maritima* and subsp. *vulgaris*, and *B*. *patula*)	1,358	2,128	0,696
**C5**	Crown of *Beta macrocarpa*	0,344	0,753	0,06
**C6**	Crown of the clade including *B*. *vulgaris* subsp. *maritima* and subsp. *vulgaris*, and *B*. *patula*	0,932	1,895	0,174

## Discussion

### Phylogenetic relationships

Our study provides new insights into the phylogenetic relationships within the Amaranthaceae family, and our major findings should help in further refinement of the taxonomy of the subfamily Betoideae. The monophyly of Betoideae was resolved with confidence in our results, but the monotypic genus *Acroglochin* was excluded from this subfamily, supporting earlier phylogenetic studies [15,16]. This genus occurs in the remote areas of the Himalayas and forms a strongly supported clade with *Corispermum chinganicum* (subfamily Corispermoideae), which is also distributed in Asian regions. Our molecular data provides evidence for the inclusion of this monotypic genus within the subfamily Corispermoideae, and is consistent with previous works (e.g. [45]). Nevertheless, we considered that further investigation is necessary to effectively test this hypothesis since there is limited taxonomic information currently available for these two genera [29].

The five extant genera of the Betoideae subfamily seem to have a relatively old origin, but their sister relationships within this clade remain unknown. The difficulty in determining the phylogenetic relationships among members of the Betoideae was evidenced by the low support for the basal nodes of this group. Indeed, our phylogenetic network reveals a rather large number of loops at the base of divergence between the five Betoideae genera (see S3 Fig). There are several reasons that may cause such an unresolved phylogenetic pattern, for example a rapid radiation of most genera that leaves little time for the accumulation of mutations and creates a substantial signal of incomplete lineage sorting [46]. Alternatively, ancient hybridization events may have occurred, which created a mosaic pattern of sequence variation. This remains to be seen when more detailed phylogenetic and population data become available, but the data presented in this study is more consistent with Kühn [14] classification, which placed the five genera in one tribe. Our results contradict those of previous works [15,16] which suggested the inclusion of the *Beta* species within the tribe Beteae, while the *Patellifolia* species were included in the tribe Hablitzieae, together with three other monotypic genera (i.e. *Aphanisma*, *Hablitzia*, and *Oreobliton*).

Even though our results cannot confidently place *Beta* and *Patellifolia* genera relative to each other, their ancient divergence reinforces their recognition as different genera, and this is supported by former morphological studies (e.g. [47]). Therefore, based on our results and previous morphological studies (M.C. Duarte, et al. unpublished data), our study indicated that *Patellifolia* species, formerly included in *Beta* section *Procumbentes* (i.e. *B*. *patellaris*, *B*. *webbiana* and *B*. *procumbens*), should be regarded as a separate genus. Together with previous molecular phylogenetic studies, our results represent a starting point for a thorough taxonomic revision of the subfamily Betoideae.

### Spatio-temporal history of Betoideae

The results of the dated molecular phylogeny suggest a relatively old origin for Betoideae, which may have taken place during the Early Oligocene Glacial Maximum (EOGM). The transition from Eocene to Oligocene was characterized by major climatic changes, which triggered extinctions in plant and animals [48]. Although the relationships among the five genera remain weakly resolved, their early diversification (ca. 32 Mya) tends to support a model of allopatric speciation within this subfamily. This could be the result of past range contractions of the most recent common ancestor of Betoideae, confirmed for instance by long branches of each genus, and reflecting possible speciation by isolation and/or extinction events during the EOGM. At least in line with such a scenario are the narrow distributions, in distant geographic regions, presented by four of the five genera, this being linked to their different ecology. Specifically, *Aphanisma* occurs in coastal habitats of California; *Hablitzia* is native in the deciduous forests of the Caucasus Region; *Oreobliton* is distributed on the chalk rocks of the Atlas Mountains in North-Africa; and *Patellifolia* is found on coastal vegetation, in maritime rocks, sea-cliffs and seashore habitats in Southern-Western Europe, with its center of diversity in the Macaronesian archipelagos. Conversely, *Beta* is the only genus of the subfamily Betoideae that presents more species diversity with a broader distribution, mainly throughout the circum-Mediterranean Region [8], and the large range observed in this genus seems to be the result of more recent climatic and geological events.

Moreover, a deep genetic differentiation between *Beta* and *Patellifolia* species, which may have occurred in the Late Oligocene was correlated with the major gene pools, reflecting an ancient divergence between *Beta* (GP1, GP2) and *Patellifolia* (GP3) species. Between the divergence of these two genera and their own diversification, there was around 15–20 Mya of uncertain evolution, considering their respective long branches, and comparing stem and crown ages of these two clades (see Fig 1). Although, our molecular data did not allow us to provide a more reliable evolutionary scenario, *Beta* and *Patellifolia* species occur in very constraining living conditions (e.g. aridity, high salinity levels), providing additional support that both lineages may have had more ability to survive the past dramatic aridity events that have occurred within the Mediterranean Region, compared to other more vulnerable plant lineages.

The second biogeographical pattern that was revealed was the occurrence of two well-differentiated clades on each side of the Mediterranean, in the western coastal areas (GP1: *B*. *vulgaris* subsp. *maritima* and subsp. *vulgaris*, *B*. *macrocarpa*, and *B*. *patula*) and in the easternmost part of the species’ distribution (GP2: *B*. *corolliflora*, *B*. *nana* and *B*. *trigyna*). The Mediterranean *Beta* species were probably beginning to differentiate around seven million years ago, which matches the Messinian Age of the Late Miocene. This coincides with the Messinian Salinity Crisis (MSC, 5.96–5.33 Mya) [49,50], a period where the connection between the Mediterranean Sea and the Atlantic Ocean closed, causing the Mediterranean Sea to desiccate and probably generating widespread salt marshes or coastal and halophytic habitats across the Mediterranean coast [50]. Such dramatic changes would have promoted the differentiation between GP1 and GP2. These two groups currently occur in different geographical areas and quite differentiated habitat types. GP1 occur in coastal cliffs, salt marshes and ruderal places of the Western Mediterranean Region and Macaronesia Islands, while GP2 is mainly present in continental mountainous zones of the Eastern Mediterranean.

This West-East disjunction pattern has also been found in other plants currently occupying the Mediterranean Basin, both in tree genera such as *Laurus* L. [51] and *Juniperus* L. [52], with representatives in Macaronesia, and in herbaceous genera such as *Erophaca* Boiss. [53]. In these studies current patterns are explained by the contraction of favorable areas, mainly due to an increase of aridity (see [53]) or by the distribution of tectonic microplates and the appearance of water barriers during the Neogene (see [51,52]). Both in the cases of *Juniperus* and *Erophaca* the authors suggest a western to eastern speciation sequence, while in *Laurus* [51], the opposite is hypothesized, with westward expansion of a single haplotype, which colonized across the Western Mediterranean, reaching the Macaronesia Islands.

The subsequent end of the MSC could additionally have promoted further differentiation by vicariance, with Western Mediterranean *Beta* populations, previously adapted to prevailing salt conditions (e.g. salt marshes habitats), being isolated by loss and fragmentation of this habitat due to post-MSC conditions. Later influential events occurred during the Plio-Pleistocene, with sea level and climate oscillations [54], leading to repeated isolation and connection of taxa, and possible subsequent speciation within the Western Mediterranean *Beta*. Some of the western wild beets would have later expanded and colonized the Macaronesian Islands. The diaspore adaptations of *Beta* and *Patellifolia* species towards sea dispersal (thalassochory) would have promoted their long-distance dispersal and have been clearly advantageous in the colonization of these archipelagos (see [55]). A key role of marine currents in dispersal was also suggested in a recent population study of *B*. *macrocarpa* and *B*. *vulgaris* subsp. *maritima*, which encompass the shoreline from France to Morocco [56]. This study suggested that *B*. *vulgaris* subsp. *maritima* went through a postglacial recolonization scenario from the Mediterranean-Atlantic region, with southern Iberia and Morocco, including the Strait of Gibraltar, acting as a long-term refuge.

Altogether, our results support the hypothesis that the Messinian Salinity Crisis and subsequent climatic changes in the Mediterranean Region during the Plio-Pleistocene were probably the major drivers of diversification in the genus *Beta*, thus explaining the current geographical ranges.

### Diversification of wild beets on Macaronesia

The estimation of divergence times provides information on the genetic distance among wild beet species, and facilitates understanding the process and timing of evolution within *Beta* and *Patellifolia* species, revealing that the diversification was quite recent, during the Pleistocene. Although this pattern was also reported for other Macaronesian native plant lineages (e.g. [57,58]), the available data does not allow us to recognize this due to insufficient phylogenetic information, and consequently we could not discard the existence of a soft polytomy, meaning that some of the *Beta* and *Patellifolia* species may have diverged at different times.

Within the *Patellifolia* clade there are unresolved polytomies and our study could not infer the monophyly of each species as well as the relationships among *P*. *procumbens* (from Madeira, Canary Islands and Cape Verde), *P*. *patellaris* (from mainland, Madeira, Canary Islands and Cape Verde) and *P*. *webbiana* (from Canary Islands) (see S2 Fig). This pattern can be the consequence of recent island colonization and differentiation, recurrent gene flow with the ancestral mainland populations or congeneric species, or even incomplete lineage sorting. Regarding the latter, the DNA regions sequenced in our study cannot provide a clear resolution for this shallow evolutionary event. Likewise, within the *Beta* clade, encompassing *B*. *vulgaris* subsp. *maritima*, the cultivated forms and all the Macaronesian species group, the phylogenetic relationships also remain unresolved. Sequences from *B*. *vulgaris* subsp. *maritima* from Madeira Island clustered with *B*. *macrocarpa* from Canary Islands, could be explained by introgression or hybridization processes, which are in accordance with the loops observed in our networks analyses (see S3 Fig). A recent study using a flow cytometry analysis, revealed the existence of mixed-ploid populations of *B*. *vulgaris* subsp. *maritima* and *B*. *macrocarpa*, in the South of Portugal [59]. Consequently our results suggest that these clustered sequences could reflect an ancient hybridization between the diploids, *B*. *vulgaris* subsp. *maritima* and *B*. *macrocarpa*, as was previously suggested by Villain [60]. This author suggested ranking the tetraploid *B*. *macrocarpa* from Canary Islands as a separate taxon, and it was proposed that these tetraploid populations result from at least two independent colonization/hybridization events in that archipelago [61]. The increased number of polyploids among these island species can be attributed to the higher adaptive potential of the polyploids [62], which might have been particularly successful in periods of ecological upheaval when new ecological niches were occupied by vigorous polyploids and less competitive diploids were outcompeted [63].

Within the *Beta* and *Patellifolia* genera, potential hybridization and the risk of demographic or genetic assimilation of rare endemics (i.e. *B*. *patula* in Madeira and *P*. *webbiana* in Canary Islands) by other native congener may occur. One possible reason is that the weakness of genetic barriers to hybridization in many islands groups is a by-product of a small genetic differentiation in recently radiated species [64]. As *B*. *patula* is classified as Critically Endangered (CR) and is one of the closest wild relatives (GP1) of domestic *B*. *vulgaris* subsp. *vulgaris*, it should be afforded higher conservation priority over the more distantly related species [2]. Thus prioritizing threatened species and conserving the entire extent of their natural ranges was recently recognized as a crucial step towards a better strategy to conserve the endemic flora in the Macaronesia archipelagos [65]. Beyond the actual or potential socio-economic value of these wild relatives as a genetic resource for crop improvement, their extinction would entail the loss of genetic resources that could help such plants overcome the future climatic shifts [66].

## Conclusions

This study uncovered the phylogenetic relationships between sugar beet (*Beta vulgaris* subsp. *vulgaris*) and the wild species, with particular emphasis on the *Beta* and *Patellifolia* species that are commonly found in coastal areas of the Western Mediterranean Region and Macaronesian Islands. The phylogeny recovered on a time-calibrated Bayesian-tree revealed a deep genetic differentiation between *Beta* and *Patellifolia* species, which may have occurred in the Late Oligocene. Furthermore, we hypothesized that ecological divergence of *Beta* in the Mediterranean Basin may have occurred during the Messinian Salinity Crisis (MSC, 5.96–5.33 Mya). Western and Eastern *Beta* species inhabit very contrasting ecological areas, from salt marshes to mountainous zones respectively. The MSC with its deep habitat modifications and extension could have provided an extraordinary period for Western Mediterranean *Beta* adaptation to these extreme ecological conditions. The subsequent end of the MSC could additionally have promoted further differentiation by vicariance due to fragmentation and isolation of a previously extended habitat. Some of the western wild beets later expanded and colonized the Macaronesian Islands during the Pleistocene. Moreover, the two endemic taxa (i.e. *B*. *patula* and *P*. *webbiana*), classified as threatened according to IUCN criteria, are associated with short phylogenetic branches and polytomic groups revealing that the diversification was quite recent in these archipelagos, and unraveling a potentially complex biogeographic pattern with hybridization and gene flow playing an important role. Finally, our phylogenetic analysis of the Betoideae sheds light on the genetic differentiation among the major gene pools of sugar beet wild relatives which are of high evolutionary, ecological, and economic relevance, providing useful data for establishing conservation priorities in the hotspot area of the Macaronesian Islands. We considered that only the conservation of populations in their natural habitats ensures renewal of gene pools and the continued supply of novel genetic material potentially critical for future crop improvement, which is recognized as an asset in maintaining global food security.

## Supporting Information

S1 Fig Distribution of *Beta* and *Patellifolia* species in the Macaronesian archipelagos (i.e. the Azores, Canaries, Cape Verde, Madeira and Salvage) and Iberian Peninsula. The size of each pie chart is proportional to the total number of studied specimens and the size of each colored sector corresponds to the proportion of sampled individuals of the corresponding taxa.
(TIF)

S2 Fig Phylogenetic relationships of the Betoideae subfamily. Maximum Likelihood (ML) tree based on ITS and cpDNA (*matK*, *trnH-psbA*, *trnL intron*, *rbcL*) concatenated dataset reconstructed using RAxML. Each operational taxonomic unit contains the species name, geographic location and sample code, respectively. Both independent runs (ML and Bayesian Inference (BI)) found essentially identical tree topologies and bootstrap values (1000 replicates) together with posterior probabilities (PP) are shown above branches (BS/PP).
(PNG)

S3 Fig NeighborNet phylogenetic network of the Betoideae subfamily based on ITS and cpDNA markers (*matK* and *rbcL*) concatenated dataset. The shaded groups correspond to: a) violet: *Patellifolia procumbens*, *P*. *patellaris* and *P*. *webbiana* (GP3); b) orange: *Beta corolliflora*, *B*. *nana* and *B*. *trigyna* (GP2) from the Eastern Mediterranean Region and Southwestern Asia; and c) green: *B*. *macrocarpa*, *B*. *patula*, *B*. *vulgaris* subsp. *maritima* and subsp. *vulgaris* (GP1) from Western Mediterranean Region and Macaronesian Islands.
(PNG)

S1 Table Description of Betoideae and Amaranthaceae vouchers and GenBank accession numbers for the corresponding ITS and cpDNA sequences. For *Beta* and *Patellifolia* samples collected for this study, the following data are provided: sampling sites, vouchers and geographic coordinates of the sampling sites.
(DOCX)

S2 Table Summary statistics for the molecular datasets. (DOCX)
