# Supplementary figures and images for: Evolutionary and Biogeographic Insights on the Macaronesian Beta-Patellifolia Species (Amaranthaceae) from a Time-Scaled Molecular Phylogeny
Source: PLoS One. 2016 Mar 31;11(3):e0152456. doi: 10.1371/journal.pone.0152456 (PMC4816301; doi:10.1371/journal.pone.0152456)

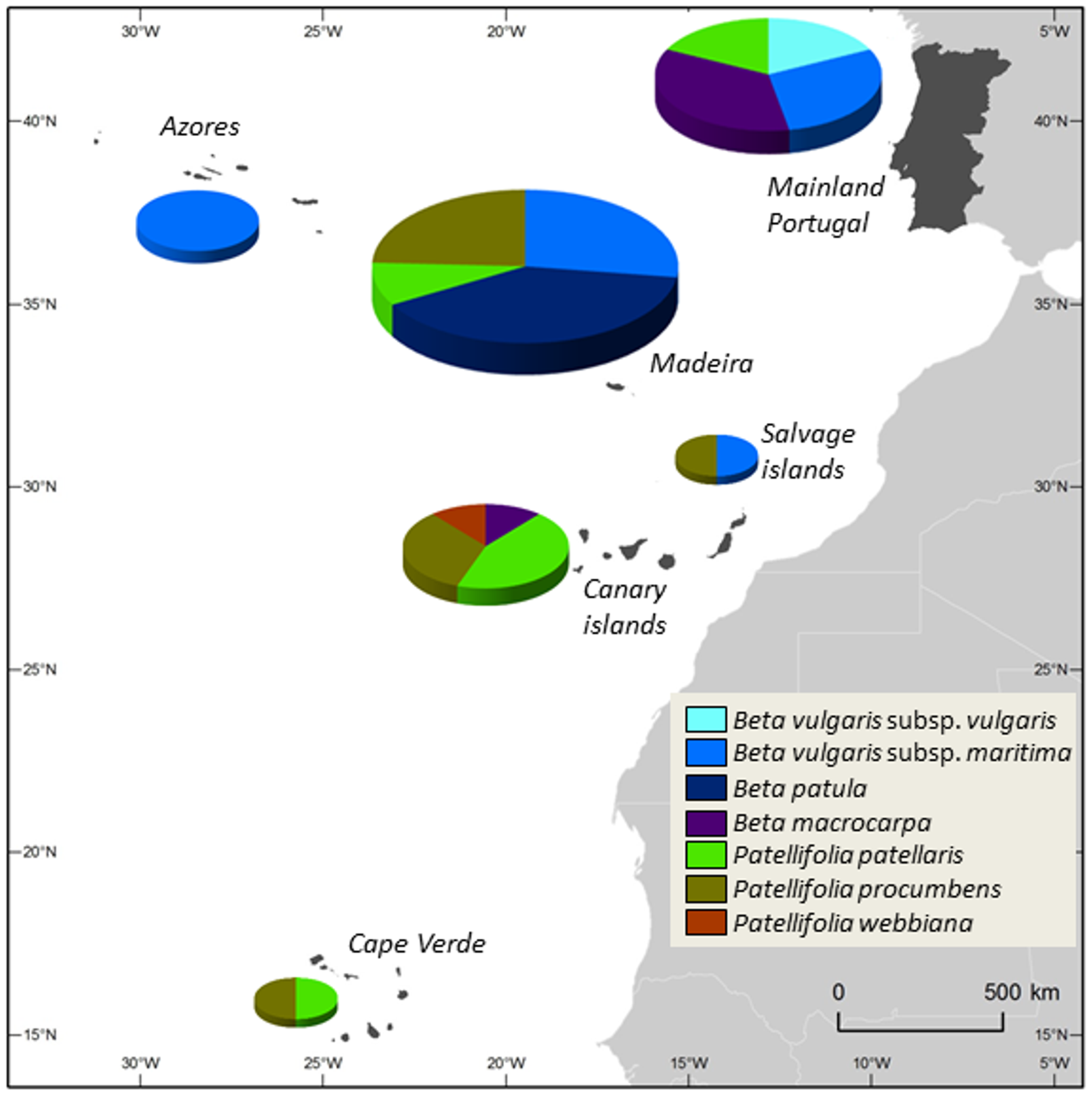

Supplement: S1 Fig — The size of each pie chart is proportional to the total number of studied specimens and the size of each colored sector corresponds to the proportion of sampled individuals of the corresponding taxa. (TIF) [file pone.0152456.s001.tif]

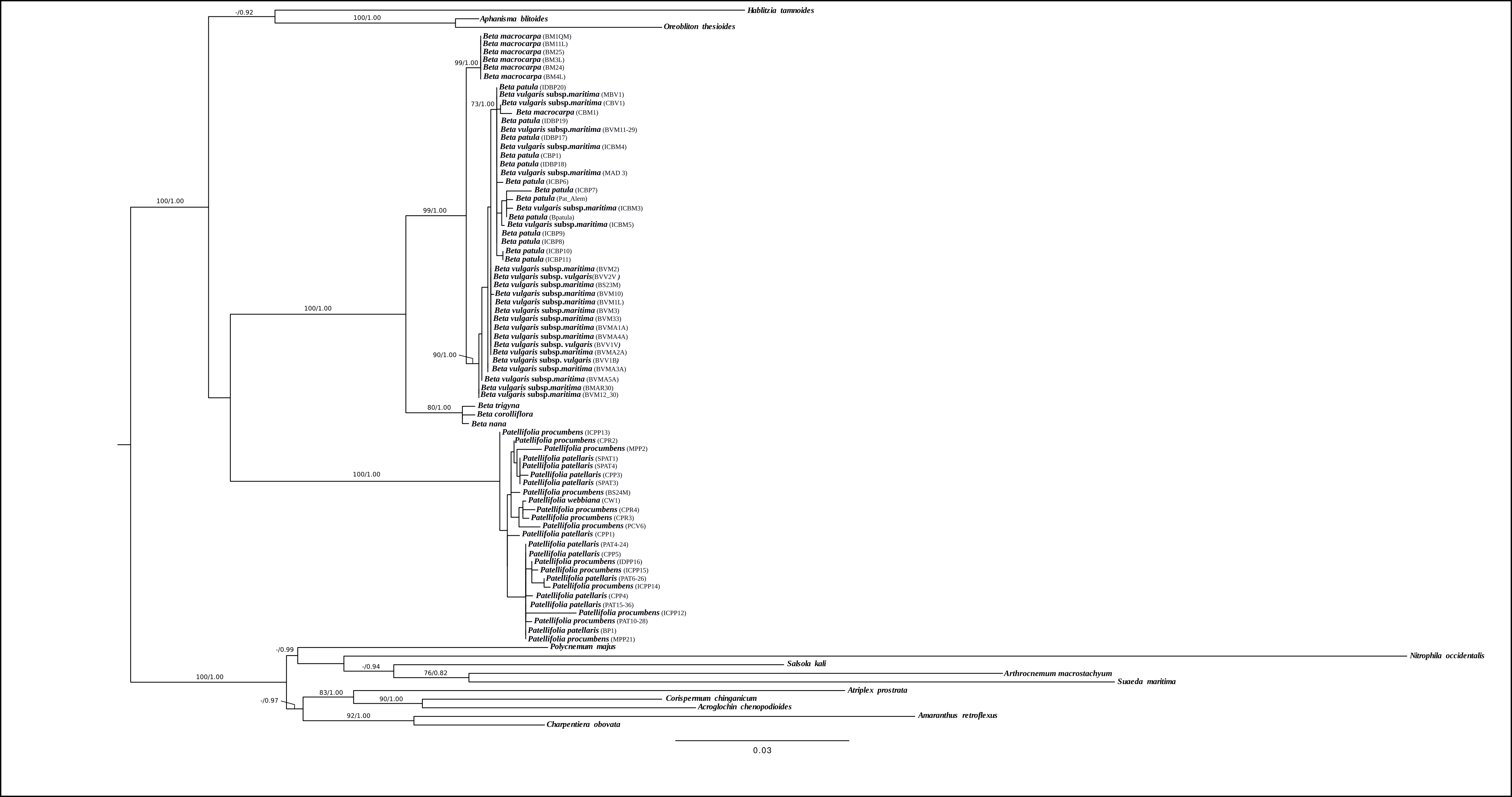

Supplement: S2 Fig — Maximum Likelihood (ML) tree based on ITS and cpDNA (matK, trnH-psbA, trnL intron, rbcL) concatenated dataset reconstructed using RAxML. Each operational taxonomic unit contains the species name, geographic location and sample code, respectively. Both independent runs (ML and Bayesian Inference (BI)) found essentially identical tree topologies and bootstrap values (1000 replicates) together with posterior probabilities (PP) are shown above branches (BS/PP). (PNG) [file pone.0152456.s002.png]

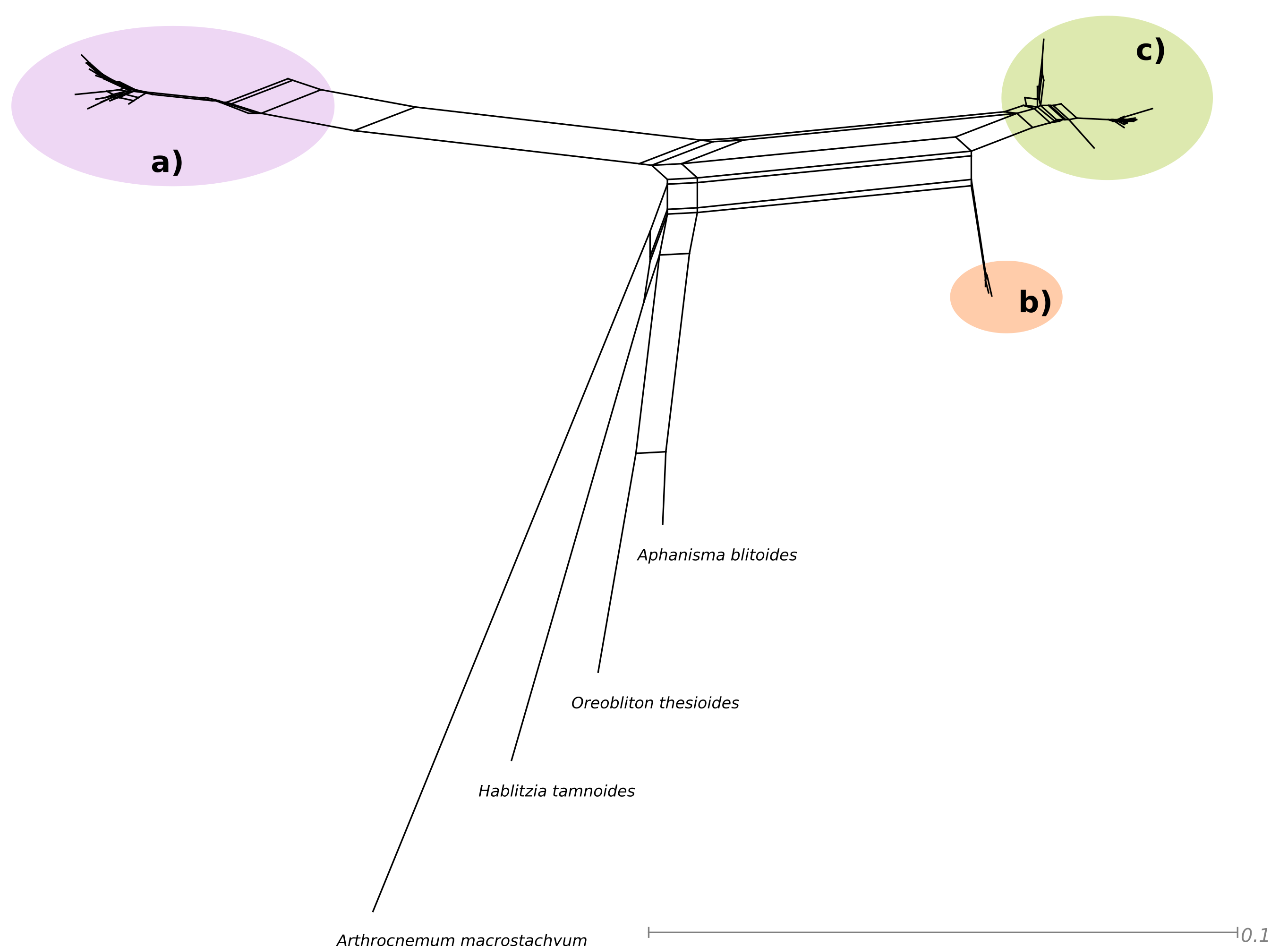

Supplement: S3 Fig — The shaded groups correspond to: a) violet: Patellifolia procumbens, P. patellaris and P. webbiana (GP3); b) orange: Beta corolliflora, B. nana and B. trigyna (GP2) from the Eastern Mediterranean Region and Southwestern Asia; and c) green: B. macrocarpa, B. patula, B. vulgaris subsp. maritima and subsp. vulgaris (GP1) from Western Mediterranean Region and Macaronesian Islands. (PNG) [file pone.0152456.s003.png]
