# Supplementary material for: Evolutionary and Biogeographic Insights on the Macaronesian Beta-Patellifolia Species (Amaranthaceae) from a Time-Scaled Molecular Phylogeny
Source: PLoS One. 2016 Mar 31;11(3):e0152456. doi: 10.1371/journal.pone.0152456 (PMC4816301; doi:10.1371/journal.pone.0152456)
Supplement: S1 Table — For Beta and Patellifolia samples collected for this study, the following data are provided: sampling sites, vouchers and geographic coordinates of the sampling sites. (DOCX) [file pone.0152456.s004.docx]

**Table 1S**. Description of Betoideae and Amaranthaceae vouchers and GenBank accession numbers for the corresponding ITS and cpDNA sequences. For *Beta* and *Patellifolia* samples collected for this study, the following data are provided: sampling sites, vouchers and geographic coordinates of the sampling sites.

| **Taxon** | **Sampling locations** | **GenBank accession numbers** | | | | | | | **Lab. Code** | **Voucher*** | **Geographic coordinates** | |
| --- | --- | --- | --- | --- | --- | --- | --- | --- | --- | --- | --- | --- |
|  |  | **ITS** | ***trnL*** | ***trnH-psba*** | ***matK*** | | ***rbcL*** | |  |  | **Latitude** | **Longitude** |
| ***SEQUENCES OBTAINED PURPOSELY FOR THIS STUDY:*** | |  |  |  | |  | |  |  |  |  |  |
| ***Beta macrocarpa*** | |  |  |  | |  | |  |  |  |  |  |
|  | Continental Portugal: Algarve, Olhão, Qtª Marim | LK054238 |  |  |  | |  | | BM1QM | M.C. Duarte 3775, 15.06.2007 | 37.0300° | -7.8194° |
|  | Continental Portugal: Algarve, Olhão, Qtª Marim | LK054234 | LK054327 | LK054353 | LK054302 | |  | | BM24 | M.C. Duarte 3774, 15.06.2007 | 37.0300° | -7.8191° |
|  | Continental Portugal: Algarve, Olhão, Qtª Marim | LK054233 | LK054326 | LK054352 | LK054301 | | LK054375 | | BM25 | M.C. Duarte 3777, 15.06.2007 | 37.0308° | -7.8155° |
|  | Continental Portugal: Algarve, Ria Formosa, Ludo | LK054235 | LK054328 | LK054354 | LK054303 | | LK054376 | | BM11L | M.C. Duarte 3796, 10.05.2009 | 37.0302° | -7.9971° |
|  | Continental Portugal: Algarve, Ria Formosa, Ludo | LK054237 | LK054342 |  | LK054305 | | LK054388 | | BM3L | M.C. Duarte 3800, 10.05.2009 | 37.0302° | -7.9974° |
|  | Continental Portugal: Algarve, Ria Formosa, Ludo | LK054236 | LK054329 | LK054355 | LK054304 | |  | | BM4L | M.C. Duarte 3798, 10.05.2009 | 37.0272° | -8.0029° |
|  | Canaries, La Palma Island, Playa Nogales | LK054277 | LK054339 | LK054365 | LK054316 | | LK054385 | | CBM1 | A. Santos s.n., 2011 | 28.75° | -17.74° |
| ***Beta patula*** | |  |  |  | |  | |  |  |  |  |  |
|  | Madeira, Desertas Islands, Ilhéu Chão | LK054247 |  |  |  | |  | | Bpatula | M.C. Duarte 3922, 07.09.2010 | 32.58° | -16.54° |
|  | Madeira, Desertas Islands, Ilhéu Chão | LK054265 |  |  |  | |  | | Icbp10 | A. Carvalho, 24.02.2009 | 32.58° | -16.54° |
|  | Madeira, Desertas Islands, Ilhéu Chão | LK054266 |  |  |  | |  | | Icbp11 | A. Carvalho, 24.02.2009 | 32.58° | -16.54° |
|  | Madeira, Desertas Islands, Ilhéu Chão | LK054262 |  |  |  | |  | | Icbp7 | A. Carvalho, 24.02.2009 | 32.58° | -16.54° |
|  | Madeira, Desertas Islands, Ilhéu Chão | LK054263 |  |  |  | |  | | Icbp8 | A. Carvalho, 24.02.2009 | 32.58° | -16.54° |
|  | Madeira, Desertas Islands, Ilhéu Chão | LK054264 |  |  |  | |  | | Icbp9 | A. Carvalho, 24.02.2009 | 32.58° | -16.54° |
|  | Madeira, Desertas Islands, Ilhéu Chão | LK054261 |  |  |  | |  | | Icbpp6 | A. Carvalho, 24.02.2009 | 32.58° | -16.54° |
|  | Madeira, Desertas Islands, Ilhéu Chão | LK054291 |  |  |  | |  | | Pat_Alem | L. Frese, 05.2010 | 32.58° | -16.54° |
|  | Madeira, Ilhéu dos Desembarcadouros | LK054278 |  |  |  | |  | | CBP1 | A. Santos, A. Carvalho, F. Fernandez s.n., 6.04.2011 | 32.73° | -16.67° |
|  | Madeira, Ilhéu dos Desembarcadouros | LK054272 |  |  |  | |  | | Idbp17 | A. Carvalho, 5.11.2008 | 32.73° | -16.67° |
|  | Madeira, Ilhéu dos Desembarcadouros | LK054273 |  |  |  | |  | | Idbp18 | A. Carvalho, 5.11.2008 | 32.73° | -16.67° |
|  | Madeira, Ilhéu dos Desembarcadouros | LK054274 |  |  |  | |  | | Idbp19 | A. Carvalho, 5.11.2008 | 32.73° | -16.67° |
|  | Madeira, Ilhéu dos Desembarcadouros | LK054275 | LK054338 | LK054364 | LK054315 | | LK054384 | | Idbp20 | A. Carvalho, 5.11.2008 | 32.73° | -16.67° |
| ***Beta vulgaris* subsp. *maritima*** | |  |  |  | |  | |  |  |  |  |  |
|  | Continental Portugal: Algarve, Ria Formosa, Ludo | LK054228 | LK054323 | LK054348 | LK054296 | | LK054372 | | BVM1L | M.C. Duarte 3795, 10.05.2009 | 37.0302° | -7.9971° |
|  | Continental Portugal: Algarve, Sagres, Cabo São Vicente | LK054229 | LK054343 | LK054346 | LK054297 | | LK054389 | | BVM33 | M.C. Duarte, J. Monjardino, A. Passarinho, I. Moura 3794, 08.08.2008 | 37.0237° | -8.9952° |
|  | Continental Portugal: Cascais, Ponta do Sal | LK054226 | LK054321 |  | LK054294 | | LK054370 | | BVM3 | M.C. Duarte, M.M. Veloso, C. Pinto-Ricardo 3757, 31.05.2007 | 38.6927° | -9.3719° |
|  | Continental Portugal: Estuário do Sado, Comporta | LK054225 | LK054320 | LK054345 | LK054293 | | LK054369 | | BVM2 | M.C. Duarte, J. Monjardino, A. Passarinho, I. Moura 3786, 25.07.2008 | 38.3834° | -8.7949° |
|  | Continental Portugal: Setúbal, Alcácer do Sal | LK054227 | LK054322 | LK054347 | LK054295 | | LK054371 | | BVM10 | M.C. Duarte, J. Monjardino, A. Passarinho, I. Moura 3787, 25.07.2008 | 38.3695° | -8.5089° |
|  | Azores, São Miguel Island, Fenais da Luz | LK054245 | LK054336 | LK054362 | LK054312 | | LK054383 | | BVMA4A | M. Romeiras & M. Moura, 23.04.2010 | 37.8283° | -25.6401° |
|  | Azores, São Miguel Island, Poças das Capelas | LK054244 | LK054335 | LK054361 | LK054311 | | LK054382 | | BVMA3A | M. Romeiras & M. Moura, 23.04.2010 | 37.8348° | -25.6758° |
|  | Azores, São Miguel Island, Poços das Calhetas | LK054243 | LK054334 | LK054360 | LK054310 | | LK054381 | | BVMA2A | M. Romeiras & M. Moura, 23.04.2010 | 37.8214° | -25.6053° |
|  | Azores, São Miguel Island, Rabo de Peixe | LK054246 | LK054337 | LK054363 | LK054313 | |  | | BVMA5A | M. Romeiras & M. Moura, 23.04.2010 | 37.8137° | -25.5862° |
|  | Azores, São Miguel Island, Santa Clara | LK054242 | LK054333 | LK054359 | LK054309 | | LK054380 | | BVMA1A | M. Romeiras & M. Moura, 23.04.2010 | 37.7346° | -25.6813° |
|  | Madeira, Desertas Islands, Ilhéu Chão | LK054258 |  |  |  | |  | | Icbm3 | A. Carvalho, 24.02.2009 | 32.58° | -16.54° |
|  | Madeira, Desertas Islands, Ilhéu Chão | LK054259 |  |  |  | |  | | Icbm4 | A. Carvalho, 24.02.2009 | 32.58° | -16.54° |
|  | Madeira, Desertas Islands, Ilhéu Chão | LK054260 |  |  |  | |  | | Icbm5 | A. Carvalho, 24.02.2009 | 32.58° | -16.54° |
|  | Madeira, Porto Moniz | LK054248 |  |  |  | |  | | Mad 3 | M.C. Duarte 3943, 17.09.2010 | 32,8681° | -17,1710° |
|  | Madeira, Porto Santo Island, Ilhéu de Ferro | LK054279 |  |  |  | |  | | CBV1 | A. Santos, A. Carvalho, F. Fernandez s.n., 6.04.2011 | 33.03° | -16.40° |
|  | Madeira, Porto Santo Island, Vila Baleira | LK054292 |  |  |  | |  | | BMAR30 | M.C. Duarte 3938, 12.09.2010 | 32.6347° | -16.9443° |
|  | Madeira, Porto Santo Island, Vila Baleira | LK054253 |  |  |  | |  | | BVM11-29 | M.C. Duarte 3931, 09.09.2010 | 33.0574° | -16.3381° |
|  | Madeira, Porto Santo Island, Vila Baleira | LK054254 |  |  |  | |  | | BVM12-30 | M.C. Duarte 3932, 09.09.2010 | 33.0574° | -16.3382° |
|  | Madeira, Praia Formosa | LK054256 | LK054344 |  | LK054314 | | LK054390 | | MBV1 | A. Carvalho, 16.10.2008 | 32.63° | -16.95° |
|  | Salvage Islands, Selvagem Grande | LK054232 | LK054325 | LK054351 | LK054300 | | LK054374 | | BS23M | M. Sim-Sim s.n., 06.08.2008 | 30.14° | -15.86° |
| ***Beta vulgaris* subsp. *vulgaris*** (cultivated forms) | |  |  |  | |  | |  |  |  |  |  |
|  | Continental Portugal: Leaf Beet (variety Blond) | LK054241 | LK054332 | LK054358 | LK054308 | | LK054379 | | BVV1B | (greenhouse) | - | - |
|  | Continental Portugal: Leaf Beet (variety Vert) | LK054240 | LK054331 | LK054357 | LK054307 | | LK054378 | | BVV2V | (greenhouse) | - | - |
|  | Continental Portugal: Sugar Beet (variety Ernestina) | LK054239 | LK054330 | LK054356 | LK054306 | | LK054377 | | BVV1V | (greenhouse) | - | - |
| ***Patellifolia patellaris*** | |  |  |  |  | |  | |  |  |  |  |
|  | Continental Portugal: Algarve, Sagres | LK054288 |  |  |  | |  | | SPAT1 | M.C. Duarte 3966, 10.12.2011 | 37.0003° | -8.9473° |
|  | Continental Portugal: Algarve, Sagres | LK054289 |  |  |  | |  | | SPAT4 | M.C. Duarte 3967a, 10.12.2011 | 37.0003° | -8.9473° |
|  | Continental Portugal: Algarve, Sagres | LK054290 | LK054341 | LK054368 | LK054319 | | LK054387 | | SPAT3 | M.C. Duarte 3967, 10.12.2011 | 37.0003° | -8.9473° |
|  | Canaries, El Hierro Island, Pozo de Sabinosa | LK054281 |  |  |  | |  | | CPP1 | A. Santos s.n., 2011 | 27.75° | -18.10° |
|  | Canaries, Fuerteventura Island, Jandía, Canarios | LK054282 |  |  |  | |  | | CPP3 | A. Santos s.n., 2011 | 28.09° | -14.27° |
|  | Canaries, Gomera Island, S. Sebastián, Pl. Avalo | LK054283 |  |  |  | |  | | CPP4 | A. Santos s.n., 2011 | 28.11° | -17.10° |
|  | Canaries, La Palma Island, Barlovento, La Fajana | LK054284 |  |  |  | |  | | CPP5 | A. Santos s.n., 2011 | 28.84° | -17.78° |
|  | Cape Verde: São Vicente Island | LK054255 |  |  |  | |  | | Pat 15-36 | L. Catarino 1805, 26.09.2010 |  |  |
|  | Madeira, Ponta de St.ª Cruz | LK054249 |  |  |  | |  | | Bp1 | M.C. Duarte 3936, 12.09.2010 | 32.6347° | -16.9443° |
|  | Madeira, Porto Santo Island, Quinta Palmeira | LK054249 |  |  |  | |  | | Pat4-24 | M.C. Duarte 3924, 09.09.2010 | 33.0667° | -16.3651° |
|  | Madeira, Porto Santo Island, Quinta Palmeira | LK054251 |  |  |  | |  | | Pat6-26 | M.C. Duarte 3926, 09.09.2010 | 33.0667° | -16.3651° |
| ***Patellifolia procumbens*** | |  |  |  |  | |  | |  |  |  |  |
|  | Canaries, Gran Canaria Island, La Isleta | LK054285 |  | LK054367 | LK054318 | | LK054392 | | CPR2 | A. Santos s.n., 2011 | 28.16° | -15.40° |
|  | Canaries, Gran Canaria Island, La Isleta | LK054286 |  |  |  | |  | | CPR3 | A. Santos s.n., 2011 | 28.16° | -15.40° |
|  | Canaries, La Palma Island, Garafía, La Fajana | LK054287 |  |  |  | |  | | CPR4 | A. Santos s.n., 2011 | 28.84° | -17.79° |
|  | Cape Verde, Santiago Island | LK054230 |  | LK054349 | LK054298 | | LK054391 | | PCV6 | J.C. Costa s.n., 09.2010 |  |  |
|  | Madeira, Câmara de Lobos | LK054257 |  |  |  | |  | | Mpp2 | A. Carvalho, 16.10.2008 | 32.64° | -16.97° |
|  | Madeira, Desertas Islands, Ilhéu Chão | LK054267 |  |  |  | |  | | Icpp12 | A. Carvalho, 24.02.2009 | 32.58° | -16.54° |
|  | Madeira, Desertas Islands, Ilhéu Chão | LK054268 |  |  |  | |  | | Icpp13 | A. Carvalho, 24.02.2009 | 32.58° | -16.54° |
|  | Madeira, Desertas Islands, Ilhéu Chão | LK054269 |  |  |  | |  | | Icpp14 | A. Carvalho, 24.02.2009 | 32.58° | -16.54° |
|  | Madeira, Desertas Islands, Ilhéu Chão | LK054270 |  |  |  | |  | | Icpp15 | A. Carvalho, 24.02.2009 | 32.58° | -16.54° |
|  | Madeira, Ilhéu dos Desembarcadouros | LK054271 |  |  |  | |  | | Idpp16 | A. Carvalho, 5.11.2008 | 32.73° | -16.67° |
|  | Madeira, Ilhéu dos Desembarcadouros | LK054276 |  |  |  | |  | | Mpp21 | A. Carvalho, 16.10.2008 | 32.73° | -16.67° |
|  | Madeira, Porto Santo Island, Vila Baleira | LK054252 |  |  |  | |  | | Patp10-28 | M.C. Duarte 3930, 09.09.2010 | 33.0581° | -16.3356° |
|  | Salvage Islands, Selvagem Grande | LK054231 | LK05432 | LK054350 | LK054299 | | LK054373 | | BS24M | M. Sim-Sim s.n., 06.08.2008 | 30.14° | -15.86° |
| ***Patellifolia webbiana*** | |  |  |  | |  | |  |  |  |  |  |
|  | Canaries, Gran Canaria Island, La Isleta | LK054280 | LK054340 | LK054366 | LK054317 | | LK054386 | | CW1 | A. Santos s.n., 2011 | 28.16° | -15.40° |
| ***SEQUENCES OBTAINED FROM GENBANK*** | |  |  |  |  | |  | |  |  |  |  |
| **Subfamily Betoideae** | |  |  |  |  | |  | |  |  |  |  |
| *Acroglochin persicarioides* | | AY858589 |  |  | AY514826 | | AY270049 | |  |  |  |  |
| *Aphanisma blitoides* | | AY858591 |  |  | AY514844 | | AY270057 | |  |  |  |  |
| *Beta corolliflora* | | AY858598 |  |  |  | |  | |  |  |  |  |
| *Beta nana* | | AY858596 |  |  |  | |  | |  |  |  |  |
| *Beta trigyna* | | AY858595 |  |  |  | |  | |  |  |  |  |
| *Hablitzia tamnoides* | | AY858590 |  |  | AY514825 | | AY270092 | |  |  |  |  |
| *Oreobliton thesioides* | | AY858592 |  |  | AY875638 | | AY270113 | |  |  |  |  |
| **OUTGROUPS** | |  |  |  |  | |  | |  |  |  |  |
| **Subfamily Amaranthoideae** | |  |  |  | |  | |  |  |  |  |  |
| *Amaranthus retroflexus* | | KF493839 |  |  | | HE967341 | | KM360629 |  |  |  |  |
| *Charpentiera obovata* | |  |  |  | | AY514855 | | AY270074 |  |  |  |  |
| **Subfamily Chenopodioideae** | |  |  |  | |  | |  |  |  |  |  |
| *Atriplex prostrata* | | HM005857 |  |  | | JX517874 | | HM849802 |  |  |  |  |
| **Subfamily Corispermoideae** | |  |  |  | |  | |  |  |  |  |  |
| *Corispermum chinganicum* | | JF792743 |  |  | |  | | JF792793 |  |  |  |  |
| **Subfamily Polycnemoideae** | |  |  |  | |  | |  |  |  |  |  |
| *Polycnemum majus* | |  |  |  | | AY514839 | | AY270118 |  |  |  |  |
| *Nitrophila occidentalis* | | FJ409841 |  |  | | AY514840 | | AY270109 |  |  |  |  |
| **Subfamily Salicornioideae** | |  |  |  | |  | |  |  |  |  |  |
| *Arthrocnemum macrostachyum* | | AY996260 |  |  | | AY996303 | |  |  |  |  |  |
| **Subfamily Salsoloideae** | |  |  |  | |  | |  |  |  |  |  |
| *Salsola kali* | | KF850551 |  |  | | HM850761 | | AY270129 |  |  |  |  |
| **Subfamily Suaedoideae** | |  |  |  | |  | |  |  |  |  |  |
| *Suaeda maritima* | | KM998343 |  |  | | KC475963 | | AY270137 |  |  |  |  |

***** Vouchers are housed in LISC, AZU, MAD, and ORT herbaria.
