# Supplementary material for: Evolutionary and Biogeographic Insights on the Macaronesian Beta-Patellifolia Species (Amaranthaceae) from a Time-Scaled Molecular Phylogeny
Source: PLoS One. 2016 Mar 31;11(3):e0152456. doi: 10.1371/journal.pone.0152456 (PMC4816301; doi:10.1371/journal.pone.0152456)
Supplement: S2 Table — (DOCX) [file pone.0152456.s005.docx]

**Table 2S.** Summary statistics for the molecular datasets.

| **Region** | **ITS** | ***trnL*** | ***trnH-psba*** | ***matK*** | ***rbcL*** |
| --- | --- | --- | --- | --- | --- |
| Number of samples | 82 | 25 | 25 | 39 | 34 |
| Alignment length (base pairs) | 713 | 580 | 366 | 2496 | 1408 |
| Sites with missing data | 379 | 88 | 248 | 1852 | 918 |
| Variable (polymorphic) sites with outgroup | 202 | - | - | 283 | 68 |
| Variable (polymorphic) sites without outgroup | 68 | 16 | 6 | 195 | 5 |
| Parsimony informative sites with outgroup | 93 | - | - | 109 | 26 |
| Parsimony informative sites without outgroup | 55 | 16 | 6 | 63 | 5 |
| Substitution model | GTR+G | HKY | F81 | GTR | GTR |
